# Resonance massage tool effects in non-migraine headache management

**DOI:** 10.22514/jofph.2025.005

**Published:** 2025-06-12

**Authors:** Gerry Leisman, Yanin Machado-Ferrer, Mauricio Chinchilla-Acosta

**Affiliations:** ^1^Movement and Cognition Laboratory, Department of Physical Therapy, University of Haifa, 3498838 Haifa, Israel; ^2^Resonance Therapeutics Laboratory, Department of Neurology, University of the Medical Sciences of Havana, 10400 Havana, Cuba; ^3^Institute for Neurology and Neurosurgery, 10400 Havana, Cuba

**Keywords:** Tension headache, Cluster headache, Resonance therapeutics, Vibration

## Abstract

**Background**: This study examines the feasibility of preventative and acute treatment of chronic cluster headaches using vibration as a potential intervention to warrant a large-scale clinical trial. **Methods**: The paper reports a study on sixty individuals suffering from tension or cluster-type headaches. The experimental group of 30 individuals received vibratory treatment at set frequencies, and the control received sham treatment. All individuals were evaluated prior to and immediately after the intervention, and at six and eight weeks after the conclusion of treatment. **Results**: Significant improvement was noted in the experimental group as rated by the Headache Impact Test-6, Rivermead Persistent Post-Concussive Syndrome (PPCS) Questionnaire, Montreal Cognitive Assessment, Participant Health Questionnaire-9, Generalized Anxiety Disorder Scale-7, and/or the Post Traumatic Stress Disorder Checklist. **Conclusions**: The use of vibration and resonance-type devices significantly reduces mean pain ratings over time, pointing to their effectiveness as a potential maintenance or preventative type of therapy. This study contributes to the development and design of larger randomized controlled trials that could further evaluate the effectiveness of vibration and resonance with oscillating expiratory pressure on headache. **Clinical Trial Registration**: ISRCTN37415803.

## 1. Introduction

Chronic headache is a significant global health concern affecting as much as 4% of the population [1]. However, most sufferers are assisted by pharmaceutical interventions, and a sizable portion cannot tolerate or show resistance to drug treatment [2, 3]. These individuals may benefit from neurostimulation therapy with peripheral or central targets [4, 5]. Numerous reports of the potential efficacy of vibratory massage or resonance-based interventions have appeared in the literature since the 1800s [6, 7, 8, 9]. The idea of treating pain with electrical or vibratory stimulation is not new. Around 46 CE (Common Era), Scribonius Largus promoted fish “electrotherapy” as a headache treatment [10]. Functional neurosurgery first relied on inducing lesions before the introduction of neurostimulation in the treatment of refractory pain [11, 12]. When neurostimulation is used, electrical or magnetic impulses are used to manipulate central or peripheral pain pathways. Its goal is to alter the pain system to lessen the intensity of pain [13, 14].

Targets for neurostimulation in headache disorders include the occipital nerve, vagus nerve, supraorbital nerve, posterior hypothalamus/ventral tegmental area, and sphenopalatine ganglion (SPG) [15, 16]. These were chosen due to the recent identification of pathophysiological pathways and proposed mechanisms relevant to animal and human models of cluster headaches [16, 17]. Central neurotransmitters can be altered by applying electrical or magnetic stimulation to pain pathways [18]. These modifications are intended as a prophylactic measure to slow down the central sensitization that develops in chronic headache [19, 20]. These modifications most likely inhibit the attack-generating mechanisms (brainstem activation or cortical spreading depression) in acute therapy [21, 22].

Existing non-invasive technologies stimulate the vagus and supraorbital nerves electrically, or the cortex magnetically [23, 24, 25]. Patients desiring to avoid, gain resistance to or who are intolerant of medication therapy may benefit from such approaches. For individuals intolerant to triptans or for whom acute medications are either ineffective or abused, devices that enable the immediate treatment of attacks may be helpful [26]. Theoretically, neurostimulation devices could be employed in a specific condition where it is difficult to use acute and preventative headache therapies, such as in pregnancy [27]. Limited open-label trials and laboratory data indicate the safety of electrical stimulation techniques in animal research, as well as transcranial magnetic stimulation and occipital nerve stimulation. Numerous studies of non-invasive means of alleviating non-migraine types of headaches have been sporadically reported in the literature that have included vibration on acupressure points [28], acoustic vibration with oscillating expiratory pressure [29] and many other such devices [30]. It is unclear how vibrational neuromodulatory technologies affect pain modulation in large measure. Previous research using oscillating expiratory pressure in conjunction with acoustic vibrations revealed improvements in both subjective and objective measures of nasal obstruction/congestion, indicating the possibility of physiological modifications in the nasal cavity as a consequence of device use. It is still unknown how these modifications affect the process by which pain is transmitted and interpreted in the trigeminal system. Previous research using sound waves in the nasal cavity has shown increases in nasal nitric oxide levels, which are known to have anti-inflammatory properties. It’s also likely that when nasal breathing is done against resistance and in the presence of acoustic energy, mechanical stimulation of the trigeminal nerve within the sinonasal mucosa downregulates pain [31].

The vagus nerve is a mixed motor and sensory nerve that regulates autonomic responses and sends signals to multiple higher centers involved in the modulation of pain [32]. The vagus nerve became a target for headache treatment after reports of migraine relief in people undergoing vagus nerve stimulation for epilepsy [33]. There is currently no evidence to support its usage in avoiding cluster headache episodes [34, 35]. This study of standard-of-care versus traditional treatment endeavors to examine the potential of preventative and acute treatment of chronic cluster headaches using a vagal nerve stimulation device.

## 2. Methods and methodology

### 2.1 Participants

A double-blind, randomized, sham-controlled, pilot study was performed for a twelve (12) week investigation. Sixty participants 18–50 years of age (M = 38.88; SD = 7.67) of whom 38 were female and 22 were male were recruited and studied from a local pain management clinic at the Institute for Neurology and Neurosurgery in Havana, Cuba (INN). Thirteen suffered from chronic cluster headaches and 37 from tension headaches. The Institutional Review Board of the INN approved the study. Written informed consent was obtained from all participants. Table 1 represents the participants included in the present study. The data collection commenced on 05 February 2023.

**Table 1.** Description of the study population. All participants were free of all drugs including over-the-counter during the course of the study.

Participant	Gender	Age	Ed. Level	Headache	Frequency/wk	Family*	Av. Duration (h)
RZX100	F	36	Univ	Tension	±3	-	±1
RZX108	M	41	Univ	Tension	±3	F	±1
RZX013	F	39	Univ	Tension	±3	M	±2
RZX162	F	40	Tech	Tension	±4	-	±1
RZX059	F	50	Univ	Tension	±5	-	±4
RZX179	F	32	HS	Tension	±3	-	±1
RZX095	M	49	Univ	Cluster	±3	-	±1
RZX102	F	36	Tech	Cluster	±3	M, MM, S	±1
RZX089	F	49	Univ	Tension	±4	-	±1
RZX148	F	28	Univ	Tension	±3	-	±1
RZX011	M	20	Univ	Cluster	±3	M, S	±1
RZX108	F	33	Univ	Tension	±5	M, S	±1
RZX026	F	39	Univ	Cluster	±3	F, B	±1
RZX210	F	37	Univ	Tension	±4	M	±2
RZX097	F	45	Univ	Tension	±7	M, S, MM	±1–1 wk
RZX138	F	28	Univ	Tension	±4	M	±1–±4 d
RZX213	F	48	Tech	Tension	±3	M, S	±2
RZX207	F	37	Tech	Tension	±3	M, MM	±1
RZX123	M	37	Univ	Tension	±3	F	±2
RZX155	F	37	Univ	Tension	±3	-	±2
RZX201	F	45	HS	Tension	±4	-	±2
RZX092	F	44	Univ	Tension	±5	M, MM	±6
RZX002	M	18	HS	Cluster	±3	F, FB	±3
RZX091	M	44	Tech	Tension	±3	M	±6
RZX007	F	18	HS	Tension	±3	M	±1
RZX035	M	40	Univ	Cluster	±8	M, MM	±2
RZX222	F	48	Univ	Tension	±7	M, S, So	±continuous
RZX199	M	32	Tech	Cluster	±3	MM	±2
RZX187	F	41	Univ	Tension	±3	-	±1
RZX196	M	33	Univ	Tension	±3	MM, MF	±1
RZX172	F	45	Univ	Tension	±3	-	±2
RZX176	F	33	Univ	Tension	±7	-	±continuous
RZX160	F	41	HS	Tension	±3	MS, MM	±2
RZX111	F	33	Univ	Tension	±3	M, MF, B	±2
RZX036	F	40	Tech	Tension	±5	M	±2
RZX133	F	48	HS	Tension	±5	M, S	±2
RZX010	F	20	HS	Tension	±3	M, MS (×3), MM	±2
RZX085	F	50	HS	Cluster	±3	F	±2
RZX053	F	50	Univ	Cluster	±7	F, B	±3
RZX050	F	45	Univ	Tension	±3	-	±2
RZX066	F	40	Univ	Tension	±3	-	±2
RZX220	M	38	Univ	Tension	±4	-	±4
RZX215	F	48	Univ	Tension	±4	M	±4
RZX129	F	48	Univ	Tension	±5	-	±2
RZX173	F	46	Univ	Tension	±4	M	±2
RZX068	M	39	Univ	Cluster	±3	F	±2
RZX077	F	37	Univ	Tension	±4	M	±2
RZX204	M	46	HS	Cluster	±16	M, F	±1
RZX194	F	37	Tech	Tension	±30	M, MM	±2
RZX218	F	38	Tech	Tension	±4	F	±2
RZX190	F	37	Univ	Cluster	±3	-	±2
RZX166	F	37	Univ	Cluster	±3	MM, MS	±1 (×2/day)
RZX115	M	38	Univ	Cluster	±3	-	±2
RZX041	F	45	Univ	Tension	±3	-	±2
RZX013	F	41	HS	Tension	±3	M, S	±2
RZX171	F	37	Tech	Tension	+5	S, D	±1
RZX074	F	37	Tech	Tension	±3	M, B	±2
RZX152	F	38	Univ	Tension	±3	-	±2
RZX229	F	37	Univ	Tension	±4	F	±2
RZX184	F	40	Univ	Tension	±7	M, S	±2

Ed.: Education; Av.: average; Univ: University; HS: High School; Tech: Technical School; *M: Mother; F: Father; S: Sister; B: Brother; MM: Mother’s mother; MF: Mother’s father; MS: Mother’s sister; So: Son; FB: Father’s brother.

### 2.2 Inclusion criteria

Participants consisted of individuals 18–50 years of age, with a diagnosis of persistent headache criteria for at least three times/week for three months to a maximum of 5 years prior to the study. The history of chronic headache is defined as ±15 days/month for >3 months. Medication use, including non-steroidal anti-inflammatories, acetaminophen, ibuprofen or aspirin excluded the participant from the experimental group. Participants suffered from either tension-type headache which presented as dull with constant pain on both sides of the head. Additional symptoms were sensitivity to light and sound, a pressure-like sensation behind the eyes, and soreness in the face, head, neck and shoulders. with episodes lasting for more than 30 minutes, or Cluster headaches. Cluster headaches are defined as severe and recurrent headaches, including other symptoms such as wet eyes, swollen eyelids, a clogged or runny nose, sensitivity to light and sound, restlessness or agitation, along with scorching or piercing pain behind or around one eye. These headaches can range anywhere from 15 minutes to 3 hours and occur unexpectedly and without notice. The attacks need to happen regularly, usually a few hours after the commencement of sleep.

### 2.3 Exclusion criteria

Excluded were individuals over 50 years of age. A history of having undergone Transcranial Magnetic Stimulation (TMS) therapy, contraindications (*e.g.*, pacemaker, metallic implant), migraine (International Classification of Headache Disorders-3 (ICHD-3)), other medical conditions (such as a history of seizures in the past or present, structural brain disease or disorders, psychotic disorders (*e.g.*, schizophrenia, bipolar disorder), liver or kidney disease, cancer, uncontrolled hypertension, diabetes, or pregnancy) were all grounds for exclusion from the study. Participants were excluded if they demonstrated a history of any neurological disease or disorder as well as allergies, sinusitis, psychiatric disorder including PTSD, anxiety, depression, fever, more than 4 cups of coffee per day, any form of medication, withdrawal from cigarettes, smoking, drugs or menstrual headache. None of the participants employed recreational drugs during the study or any other chronically employed drugs. Non-steroidal anti-inflammatory drugs may be associated with headache despite their efficacy, studies have noted that their use in acute pain episodes can prolong pain and inflammation delay ameliorating the pain, and potentially interfere with the results [36].

Also excluded were individuals suffering from headaches other than cluster or tension-type headaches including migraine defined clinically as individuals who at the outset of a headache manifested symptoms that included partial loss of vision, numbness, tingling and muscle weakness; sensitivity to light, sound and smell; nausea and/or vomiting; auras present before the headache appears, with or without the presence of zigzag lines, flashing lights or spots; or trouble speaking or finding words (dysnomia). Also excluded were those with hypnic headaches that usually commence when individuals are over fifty years of age, although they can begin sooner. They have been referred to as “alarm clock” headaches, as they can awaken individuals at night. Excluded was anyone waking in the night from a headache. Mild to severe throbbing pain, generally in both sides of the head, is a principal symptom of a hypnic headache. It can linger for up to three hours, and other symptoms including light and sound sensitivity and nausea are possible. Medication-overuse headache, sinus headache (occurring with sinusitis) accompanied by a throbbing, dull pain radiating over the forehead, cheeks and eyes; face pain or pressure; nasal discharge and plugged nose. Caffeine-related headaches were excluded with a high caffeine intake of greater than 400 mg, or around 4 cups of coffee per day. Head Injury or post-Head Injury headaches, menstrual headache and hangover headache were likewise excluded.

### 2.4 Clinical assessments

Demographic information was collected 2–4 weeks before starting the study, comprising social and family medical history, medication use, past medical history, age, sex, education and allergies. The frequency, intensity, medication use, type of headache, accompanying symptoms (such as neck pain, photophobia, phonophobia, nausea or vomiting) and headache triggers were all included in the headache history.

We defined a change in headache frequency or severity at 1 month post-treatment as the primary outcome. Participants created a 2-week headache journal before, during, and after resonance treatment. We also conducted follow-up assessments at the first and third post-treatment start, for a total of 12 weeks of diary entries. Headache frequency was documented as a headache being present in the morning, afternoon, or evening each day. A maximum of 42 headaches per week was possible by adding these frequencies together. The Numeric Pain Rating Scale (NPRS), an 11-point scale from 0 to 10, was used to measure the intensity of headaches. A score of 0 denoted no pain, while a score of 10 denoted the most severe agony that can be imagined. Only in cases where a headache was present was severity recorded.

The Headache Impact Test-6 (HIT-6), Rivermead PPCS Questionnaire (RPSQ), Montreal Cognitive Assessment (MoCA), Participant Health Questionnaire-9 (PHQ-9), Generalised Anxiety Disorder Scale-7 (GAD-7) and/or the Post Traumatic Stress Disorder Checklist for DSM-5 (PCL-5) were among the baseline questionnaires used to assess secondary outcomes. After their treatment (day 60) and one month later, the participants underwent another assessment. Every follow-up appointment included the completion of the questionnaires.

### 2.5 Procedure

The initial study was conducted using the Rezzimax® Tuner Pro II (Rezzimax, Richmond, UT, USA) device with a tuning fork attachment represented in Fig. 1 and whose physical characteristics are appended in **Supplementary Fig. 1**. It is a battery-operated resonant massage tool. The device has a frequency range of between 20 and 120 Hz with 10 preset levels. It also has 4 proprietary algorithms or patterns to assist in decreasing pain. For this study, the patterns were not utilized. Participants selected a comfortable level between 1 and 10 for each technique.

**Fig. 1.** Rezzimax® Tuner Pro II device with a tuning fork attachment.

Each of the participants received the following instructions. Each participant placed their tongue between their teeth. They were then instructed to hum with the resonance of the Rezzimax® Tuner Pro II, illustrated in Fig. 1. The procedure is described more fully at the following link (https://www.youtube.com/watch?v=iyFZ79aw4Lk). The tuner and the tong were used at a variable level of between 5 and 10. Tongs were placed on both sides of the neck with the tuner device resting against the spine. The tuner was held in place by the back of the neck keeping it in place. The participant was requested to rotate their head from side to side maintaining pressure against the tuner. The device was kept in place for two minutes with a pillow behind the neck pressing against the tuner. An intensity level of 6 or lower was applied and the tongs were applied over the central portion of each eyebrow. After two minutes the tongs were then placed on the top of the nose towards the forehead. The tuner was then kept in place for an additional two minutes. The intensity was increased up to level 10 or lower if uncomfortable to the individual. Afterward, the tongs were placed under the jaw with the tuner held in place with both hands by the participant. After this stage, the tongs were covered in a plastic cover and placed inside of the mouth between the cheeks and teeth. The tuner was then turned from side to side for one minute and then the participant was required to open and close the moth with the tuner in place for an additional one minute.

### 2.6 Apparatus

The Rezzimax® Tuner Pro II device contained a Precision Microdrive Model No. 320-102 vibration motor with a rated operating voltage of 3 V and rated vibrational speed of 790 rpm (+/−1600) and a normative amplitude of 17 G. The DC motor characteristics can be found in the appended **Supplementary Fig. 1**.

### 2.7 Randomization and blinding

Randomization for participant allocation to two groups was executed. The groups were: Experimental (E_NS_) receiving the vibration treatment and Control A (C_S_) receiving sham intervention. Randomization was performed via a randomized block design with varying block sizes of two, four and six participants. In each block, one-half of the participants were randomly assigned to Group C_S_, and another half to Group E_NS_. Using computer-generated sequence methods, randomization was achieved while maintaining participant and investigator confidentiality about both the generated allocation sequence and the randomization approach.

Since each computer-generated randomization sequence was distinct, replication was impossible. Randomization was applied to “Group E_NS_” or to “Group C_S_”. Only the designated individual at the study site knew which assignment corresponded to which experimental-treatment or control group, with this information not to be revealed until study unblinding occurred, after all data had been entered into the database, and the database sealed before statistical analyses.

### 2.8 Statistical analysis

This study examined a sample of 60 individuals (30 in Group E_NS_ and 30 in C_S_), who were recruited at the Institute for Neurology and Neurosurgery, headache, and chronic pain programs, as well as poster advertisements in hospital clinics. Descriptive statistics and frequency distributions were used to evaluate the baseline sample characteristics.

Results were considered statistically significant with a value of <0.05. In cases where a significant group-by-time interaction was found, simple effects testing with a Bonferroni correction was performed. The blinding’s integrity was evaluated using a chi-square test. Biostatisticians provided advice during the analyses, which were carried out using SPSS software (v. 25; SPSS, Inc., IBM, Chicago, IL, USA). Requests for individual participant deidentified data are available at doi: 10.13140/RG.2.2.18953.45920.

## 3. Results

The results of the study are reported in Table 2 with tests of normality applied to the data as described below.

**Table 2.** Tests of normality were applied to data recorded at the outside of the experiment and the end, then six and eight weeks later.

Tests of Normality							
		Kolmogorov-Smirnov^a^			Shapiro-Wilk		
Test session		Statistic	*df*	Sig.	Statistic	*df*	Sig.
PRE							
	GAD-7	0.270	89	N.S.	0.552	89	N.S.
	HIT-6	0.111	89	0.009	0.924	89	N.S.
	MoCA	0.465	89	N.S.	0.309	89	N.S.
	NPRS	0.169	89	N.S.	0.915	89	N.S.
	PHQ-9	0.211	89	N.S.	0.622	89	N.S.
END							
	GAD-7	0.118	89	0.004	0.952	89	0.003
	HIT-6	0.137	89	N.S.	0.956	89	0.004
	MoCA	0.382	89	N.S.	0.615	89	N.S.
	NPRS	0.117	89	0.004	0.953	89	0.003
	PHQ-9	0.087	89	0.094	0.958	89	0.006
SIX							
	GAD-7	0.127	89	0.001	0.959	89	0.007
	HIT-6	0.123	89	0.002	0.940	89	N.S.
	MoCA	0.477	89	N.S.	0.387	89	N.S.
	NPRS	0.105	89	0.017	0.958	89	0.006
	PHQ-9	0.086	89	0.102	0.959	89	0.006
EIGHT							
	GAD-7	0.105	89	0.017	0.934	89	N.S.
	HIT-6	0.148	89	N.S.	0.926	89	N.S.
	MoCA	0.472	89	N.S.	0.338	89	N.S.
	NPRS	0.128	89	0.001	0.942	89	0.001
	PHQ-9	0.105	89	0.017	0.964	89	0.015

*^a^Lilliefors Significance Correction. PRE: Prestesting; END: after 60 days; SIX: after 74 days; EIGHT: after 88 days; GAD-7: Generalised Anxiety Disorder Scale-7; HIT-6: Headache Impact Test-6; MoCA: Montreal Cognitive Assessment; NRPS: Numeric Pain Rating Scale; PHQ-9: Participant Health Questionnaire-9; N.S.: not significant.*

The results reported in Table 2 illustrate the testing of normality assumption results with a Lilliefors significance correction applied to the Kolmogorov-Smirnov test and supported by the Shapiro-Wilk test. The Kolmogorov-Smirnov (K-S test) is a nonparametric test of the equality of continuous (or discontinuous) one-dimensional probability distributions used to compare two samples (two-sample K-S test). “How likely is it that we would see two sets of samples like this if they were drawn from the same (but unknown) probability distribution”? The null hypothesis of the Wilkes-Shapiro test is that the population is normally distributed. Thus, if the *p*-value is less than the chosen alpha level, then the null hypothesis is rejected and there is evidence that the data tested are not normally distributed. Conversely, the null hypothesis (that the data originated from a regularly distributed population) was not rejected if the *p*-value exceeded the selected alpha threshold. Therefore, it was concluded that the data set was not from a normally distributed population.

When tested statistically in an aparametric test in the L*K square, a significant difference was indeed found in the experimental group compared to the control group in all scales as reflected in Fig. 2A,B.

**Fig. 2.** Pre-post treatment effects. (A) Reflects the results comparing pre- versus post-treatment on the five scales measured that include the GAD-7, HIT-6, MoCA, NPRS and PHQ-9; (B) Demonstrates no significant differences for the control group on all of the five measures between pre-and post-testing. GAD-7: Generalised Anxiety Disorder Scale-7; HIT-6: Headache Impact Test-6; MoCA: Montreal Cognitive Assessment; NRPS: Numeric Pain Rating Scale; PHQ-9: Participant Health Questionnaire-9.

If we examine Table 3 as well as Fig. 3, we see that there is no significant difference between the condition of the experimental group and the control group before the experiment (PRE) and eight weeks after (EIGHT). On the other hand, when we look at the group that finished the treatment (END) compared to the control group and when we look at the same group after 6 weeks of the treatment (SIX) compared to the control, we see a significant difference *p* < 0.001. The statistical test is a non-parametric Kruskal-Wallis because the data are not normally distributed.

**Table 3.** Kruskal-Wallis Test results at six and eight weeks after the conclusion of the study showed significant differences in headache pain perception comparing pre and post-testing.

Test Statistics^a,b^				
	PRE	END	SIX	EIGHT
Kruskal-Wallis H	0.237	19.119	10.506	1.242
*df*	1	1	1	1
Asymp. Sig.	0.626	N.S.	0.001	0.265

*^a^Kruskal-Wallis Test; ^b^Grouping Variable: GA_SPLIT; PRE: Prestesting; END: after 60 days; SIX: after 74 days; EIGHT: after 88 days; N.S.: Not significant; Asymp. Sig.: two-tailed significance/p-value.*

**Fig. 3.** Significance of difference in integrated pain perception testing at the study’s outset, at the conclusion of the study, six and eight weeks later comparing groups. (A) (E_NS_ Treatment) and (B) (C_S_ Sham treatment).

## 4. Discussion

It has been shown that cutaneous vibration can reduce symptoms in those with chronic pain [37], osteoarthritis [38] and muscle pain [39]. By triggering a pain gating system in the brainstem, cutaneous vibration modifies pain by influencing mechanoreceptors in the face [40]. Although vibration has been used to lessen facial pain from injections [36] and transcutaneous electrical stimulation has been shown to relieve headaches [41], headache patients have not been evaluated with cutaneous vibration.

It has been proven that 1-MHz frequency waves can relieve pain in headache patients [42]. Deep tissue temperatures are raised by these high frequency waves by 1–5 °C [43, 44]. Additionally, high-frequency waves may lessen inflammation [45]. Targeting both deep and surface mechano- and thermosensitive nerves, cutaneous vibration may more successfully reduce pain brought on by some forms of headaches.

Precise pain measurements are necessary to determine clinically substantial pain relief. The placebo effect is very strong in pain trials, which raises the need for exact measurements. The questionnaires used in this study have been validated for use in pain studies across a variety of demographics, but they have not been used to examine the effects of vibration massage-type pain treatment.

The stimulation of mechanosensors by cutaneous vibrational massage and how it might work as an analgesic was our main theory. Each patient received treatment using a specialized multimodal vibration therapy device made by Rezzimax® that has a transducer head that is tailored to the geometry of the face.

The usage of Rezzimax® seems to reduce mean pain ratings over time, pointing to its effectiveness as a maintenance or preventative type of therapy. In the end, this study will contribute to the development and design of a larger randomized controlled trial that will further evaluate the effectiveness of the vibration and resonance with oscillating expiratory pressure on headache.

Inadequate consultation with a qualified healthcare provider, inability to reach a definitive diagnosis and inadequate use of suitable acute and preventive medication are recognized obstacles to headache treatment [46]. Patients who receive medical treatment for headache disorders may have a variety of adverse effects depending on the medicine. Triptans can cause drowsiness, dry mouth, muscle weakness, and vertigo as side effects. Triptans are not recommended for people who have these conditions since they have been connected to heart attacks and strokes, though very rarely [47, 48].

When analyzing the findings of this study, there are several limitations. First off, participants exhibiting objective signs of a headache other than a tension and cluster headache were expressly excluded from this study. Therefore, it is impossible to predict whether patients with comorbid or other forms of headache would see a comparable level of efficacy. Additionally, outcomes cannot be completely free from the influence of placebo effects and regression to the mean. Also, no significant differences were found in the results between tension and cluster headaches, and as a result, the data was pooled. Since the number of participants was too small to include types of headaches as nested factors, study results could not be adequately compared across various types of headaches. Although the exact mechanisms of action of vibratory-massage devices are unknown, we can speculate based on earlier research that as significant improvements in the objective and subjective metrics of nasal congestion/obstruction were found when similar devices were used to treat headache, physiologic changes within the nasal cavity, may have occurred in response to device use [29]. Earlier research on acoustic energy applied to the nasal cavity showed increases in nasal nitric oxide, which may also modify the pain pathway through anti-inflammatory effects [29]. However, we do not know the mechanisms of action of the effect. The next logical step in assessing this form of therapy is to perform more studies that include suitable control groups with numerous forms of headache. The final limitation was the shorter follow-up time (only 8-week), and thus future research may examine effectiveness over a longer time frame and/or examine the usefulness of different regimens, such as use only when necessary.

## 5. Conclusions

The use of vibration and resonance-types devices significantly reduces mean pain ratings over time, pointing to its effectiveness as a potential maintenance or preventative type of therapy in headache management.

## Supplementary Material

Supplementary material associated with this article can be found, in the online version, at https://files.jofph.com/files/article/1899699815561740288/attachment/Supplementary%20material.docx.


