# Supplementary material for: Resonance massage tool effects in non-migraine headache management
Source: J Oral Facial Pain Headache. 2025 Jun 12;39(2):84–93. doi: 10.22514/jofph.2025.005 (PMC12520439; doi:10.22514/jofph.2025.005)
Supplement: Supplementary file 1 [file Supplementary-material.docx]

Supplementary material


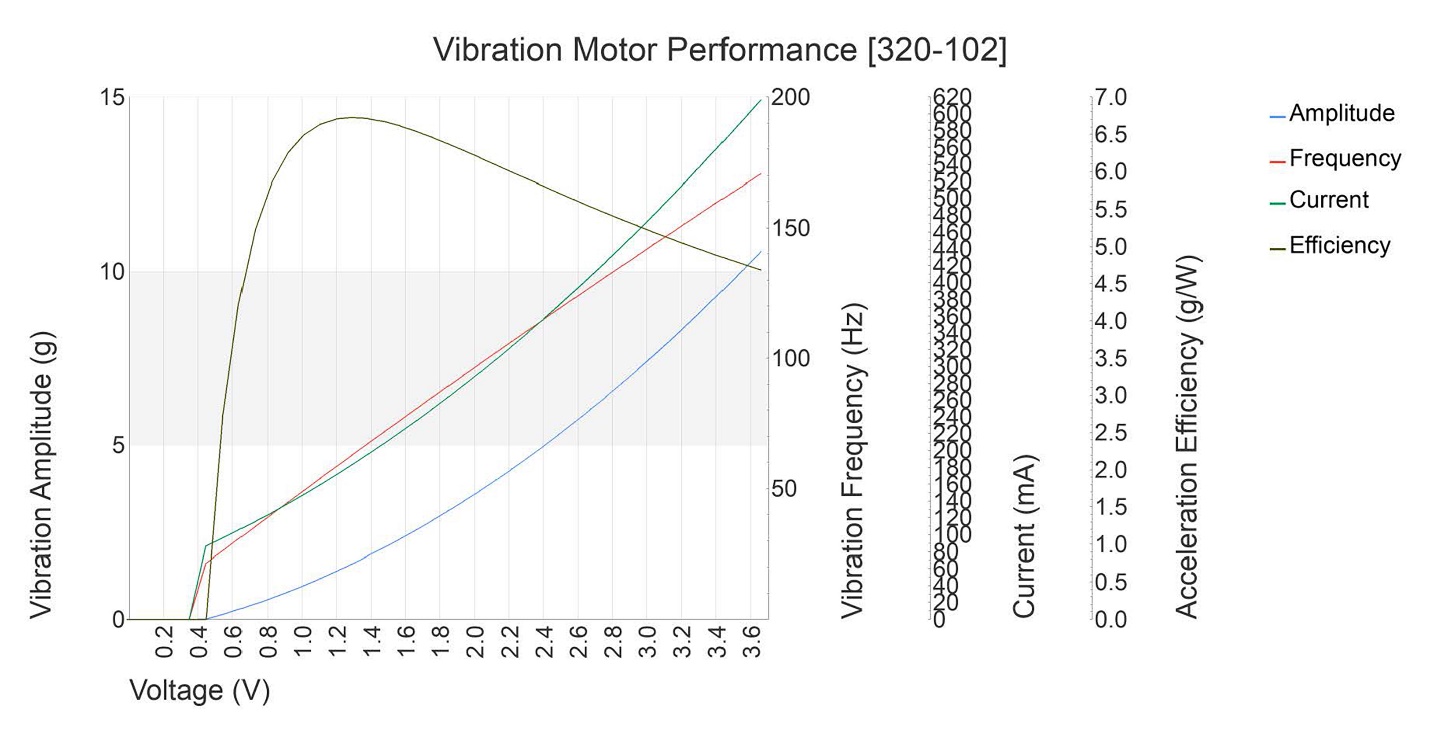


Supplementary Fig. 1. Vibration motor performance characteristics of the precision Microdrive 320–102.
